# Sensor Measurements Can Characterize Fluctuations and Wearing Off in Parkinson’s Disease and Guide Therapy to Improve Motor, Non-motor and Quality of Life Scores

**DOI:** 10.3389/fnagi.2022.852992

**Published:** 2022-03-23

**Authors:** Parisa Farzanehfar, Holly Woodrow, Malcolm Horne

**Affiliations:** ^1^Parkinson’s Laboratory, Florey Institute of Neurosciences and Mental Health, Parkville, VIC, Australia; ^2^Department of Clinical Neurosciences, St. Vincent’s Hospital Fitzroy, Fitzroy, VIC, Australia

**Keywords:** Parkinson’s disease, fluctuations, wearing off, objective measurements, wearable sensors, motor complications in Parkinson’s disease

## Abstract

**Objectives:**

The aim was to examine the role of sensor measurement in identifying and managing fluctuations in bradykinesia of Parkinson’s Disease.

**Method:**

Clinical scales and data from wearable sensors obtained before and after optimization of treatment from 107 participants who participated in a previous study was used. Fluctuators were identified by a levodopa response or wearing off in their sensor data and were subdivided according to whether the sensor’s bradykinesia scores were in target range, representing acceptable bradykinesia for part of the dose (Controlled Fluctuator: *n* = 22) or above target for the whole dose period (Uncontrolled Fluctuator; *n* = 28). Uncontrolled Non-fluctuators (*n* = 24) were cases without a levodopa response or wearing-off and sensor bradykinesia scores above target throughout the day (un-controlled). Controlled Non-fluctuators (*n* = 33) were below target throughout the day (controlled) and used as a reference for good control (MDS-UPDRS III = 33 ± 8.6 and PDQ39 = 28 ± 18).

**Results:**

Treating Fluctuators significantly improved motor and quality of life scores. Converting fluctuators into Controlled Non-fluctuators significantly improved motor, non-motor and quality of life scores and a similar but less significant improvement was obtained by conversion to a Controlled Fluctuator. There was a significantly greater likelihood of achieving these changes when objective measurement was used to guide management.

**Conclusions:**

The sensor’s classification of fluctuators bore a relation to severity of clinical scores and treatment of fluctuation improved clinical scores. The sensor measurement aided in recognizing and removing fluctuations with treatment and resulted in better clinical scores, presumably by assisting therapeutic decisions.

## Introduction

In the first few years of Parkinson’s Disease (PD), bradykinesia responds well to levodopa and other dopaminergic medications (Fahn et al., 2004; Lees et al., 2009). Nevertheless, within 2 years of diagnosis, approximately 50% of people with PD (PwP) become aware of progressive shortening of benefit from levodopa doses (Ahlskog and Muenter, 2001). This phenomenon, referred to as “wearing-off,” is experienced eventually by around 70% of PwP. Wearing-off is preceded by a response to levodopa, which can be measured by a levodopa challenge test (Chou et al., 2018). The clinical assessment and classification of these transitions between the levodopa response and the “off” state have been comprehensively reviewed (Chou et al., 2018) and are referred to here as “fluctuations.” PwP do not always perceive wearing-off as re-emergence of bradykinesia (Stacy, 2010; Matthews et al., 2015) but may experience transitions to non-motor symptomologies (Matthews et al., 2015; Chou et al., 2018) and self-awareness of bradykinesia may be limited in some cases (Maier and Prigatano, 2017). Consequently, treating clinicians are frequently unaware of the presence of “wearing-off” (Martinez-Martin and Hernandez, 2012; Erb et al., 2020).

As the development of fluctuations significantly reduces quality of life of PwP (Dodel et al., 2001; Rodriguez-Violante et al., 2018), their accurate recognition presents opportunities to improve quality of life for PwP (Farzanehfar et al., 2021). Wearable devices are now available for routine care of PwP (Farzanehfar et al., 2018; Woodrow et al., 2020) and offer a possible aid in detecting and assessing fluctuations (Erb et al., 2020; Farzanehfar et al., 2021; Khodakarami et al., 2021). The feasibility of using sensors to measuring fluctuations has been demonstrated (Farzanehfar et al., 2018; Khodakarami et al., 2019, 2021; Erb et al., 2020; Woodrow et al., 2020) and one of these sensor systems, the Parkinson’s KinetiGraph (PKG, Global Kinetics Corporation™, Australia), was used in this study.

Several requirements of sensor systems, over and above the ability to measure bradykinesia, are necessary for the detection and assessment of fluctuations in clinical care. These include data points that are frequent enough to measure clinically relevant changes in dopaminergic transmission, measurement over a long enough period to capture the response to every dose on several occasions and measurement that does not require interruptions to daily activities. Any sensor system that met these requirements could have been used in this study. To our knowledge the PKG system is the only one that meets all these requirements. A particular requirement that is not dependent on the technology is a defined target range. The idea of a target range is widespread in medicine, and in the case of PD, it would be the boundary separating acceptable or unacceptable states of bradykinesia. In the case of the PKG, targets were recommended by an expert panel (Odin et al., 2018) and approximates to a Movement Disorder Society- Unified Parkinson’s Disease Rating Scale (MDS-UPDRS III) score in the high twenties. A similar target was used in earlier studies (Farzanehfar et al., 2018; Woodrow et al., 2020) and resulted in an average MDS-UPDSR III score of 28.6 when used as a treatment target (Woodrow et al., 2020).

Two further points are relevant to measuring fluctuations with sensors. The first is that usual clinical assessment requires the PwP to recognize increasing bradykinesia as a manifestation of fluctuations (Hauser et al., 2004; Stacy, 2010; Antonini et al., 2011). This is reporting of a symptom, whereas objective measurement of bradykinesia by sensors provide an objective measurement of change in the level of bradykinesia regardless of the subject’s perception. Consequently, the symptomatic reporting of fluctuations may not be well correlated with their objective measurement (Ossig et al., 2016). The question being addressed here is not how these two methods concur but whether objective measurement would aid clinical management, and this is most directly tested by comparing outcomes when the objective measurement is available with conventional clinical assessment.

The second point is that recording a PwP’s perception of transition to the “off” state (for example in a diary) does not require comment on whether the best or “on” state is the lowest attainable bradykinesia: this method recognizes a levodopa response but not whether that response reduced bradykinesia to an acceptable objective level. With objective measurement both these assessments must be made (see section “Materials and Methods” for a detailed discussion): is a levodopa response present and does the response achieve a satisfactory reduction in bradykinesia. Thus, for a participant to be labeled a “fluctuator” using objective measurement requires a levodopa response to be seen in the recorded data. Knowing the duration of that response is also therapeutically useful. The second assessment needed in objective measurement is whether the response has reduced bradykinesia to an acceptable level without excess dyskinesia (i.e., in target). If the best response is an acceptable level of bradykinesia but the dose interval is too long resulting in wearing-off to an unacceptable level of bradykinesia, then shortening the dose interval will eliminate the wearing-off while avoiding an increase in dose that might induce peak dose dyskinesia. On the other hand, a levodopa response that did not sufficiently reduce bradykinesia (to target) will require an increase in dopaminergic transmission and may also require shorter dosing intervals. In other words, there is a therapeutic target defined by objective measurement scores, and effective treatment is when the levodopa response results in bradykinesia scores being in target and the timing of subsequent doses is such that wearing-off does not result in re-emergence of bradykinesia outside of this target range or result in dyskinesia.

The aim of this study is to use objective measurements to identify fluctuations and guide the adequacy of treatment and to assess the extent that this improves clinical scales and scores related to quality of life. The explicit point of the above discussion is that the information from the sensor system must lead to useful therapeutic decisions. A direct means for comparing the value of sensor information is to comparing the outcomes of fluctuators managed using information gained in the usual clinical way with the management of fluctuators where information from a sensor system was also available. A previous study designed along these lines was directed at the over-all management of PD (Woodrow et al., 2020). Its aim was to assess the value of sensors on a cohort whose eligibility was mainly determined on having no contraindications to increasing the dose of dopaminergic agents and did not address or compare the response to fluctuators. The study showed that there was a significant greater improvement in MDS-UPDRS III and Total Scores when sensor data was available. PDQ-39 scores also improved when those participants treated for bradykinesia were analyzed separately (the majority). However, this study did not comment on how fluctuators were affected. This data was re-analyzed to first assess whether the classification of fluctuators using sensors (outlined above and in section “Materials and Methods”) resulted in meaningful clinical distinctions, secondly did the classification and clinical scores change following treatment and did the availability of the PKG information at the time of treatment affect the outcome.

Previous studies show the feasibility of measuring fluctuations using wearable sensor such as the PKG (Farzanehfar et al., 2018; Khodakarami et al., 2019, 2021; Erb et al., 2020; Woodrow et al., 2020), which classifies fluctuations according to whether the levodopa response was sufficient to reach target and was maintained with (or without) wearing-off (Woodrow et al., 2020; Khodakarami et al., 2021).

## Materials and Methods

### The Parkinson’s KinetiGraph System

**FIGURE 1:**
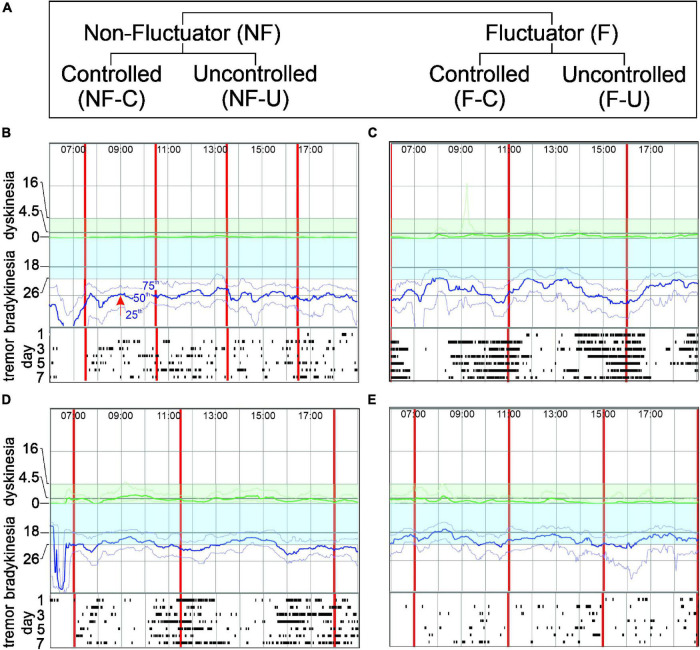
Panel **(A)** shows the Fluctuator classification. Panels **(B–E)** show PKG excerpts representing each classification. In each PKG: X axis- time of day (top); vertical red lines-time of each dose; Y axis-BKS and DKS scales. Bradykinesia target region is shaded blue. Moving BKS_50_ [red arrow, Panel **(B)** shows moving BKS_50_ at 9:00] is a heavy blue line: moving BKS_25_ and moving BKS_75_ are lighter blue lines above and below moving BKS_50_ **(B)**. Rasters below each graph show tremor: each line represents a day and dots indicating 2-min epochs when tremor was present. DKS severity increases from zero to top of graph and target region is shaded green. Moving DKS_50_ is a heavy green line and moving DKS_75_ and moving DKS_25_ are lighter green lines above and below (respectively). **(B)** Non-fluctuator Uncontrolled (NF-U). Moving BKS unchanged following levodopa doses (Non-fluctuator) and lies outside the target range all day (Uncontrolled) and tremor does not vary with dose. **(C)** Fluctuator Uncontrolled (F-U). Moving BKS falls following each levodopa doses (Fluctuator) but even at peak response is above target range on most days (Uncontrolled). Tremor reduces with each dose. **(D)** Fluctuator Controlled (F-C). Moving BKS falls following each levodopa doses (Fluctuator) to enter target range for 2–3 h (thus “controlled”) and then leaves target when dose “wears-off”. Tremor reduces with each dose. **(E)** Non-fluctuator Controlled (NF-C). Moving BKS is within target all day (Controlled) even though it varies with each dose.

The PKG system consists of a wrist-worn data logger, a series of algorithms that produce data points for bradykinesia (Griffiths et al., 2012) and dyskinesia (Griffiths et al., 2012) every 2 min of recording (epoch) and a report (or PKG), which plots these 2-min scores against the time of day (Figure 1; Griffiths et al., 2012; Kotschet et al., 2014; McGregor et al., 2018; Khodakarami et al., 2019, 2021). Data is typically collected for 6 days, and relevant parameters are detailed in the following Glossary.

#### Glossary of Parkinson’s KinetiGraph Terms

BKS: Accelerometry data from each 2 min epoch is analyzed to produce a bradykinesia score (BKS) (Griffiths et al., 2012). BKS > 80 indicate sleep (McGregor et al., 2018) and 80 > BKS > 40 indicates inactivity.

Median BKS: The median of BKS < 80 (i.e., sleep and “off wrist” excluded) between 09:00 and 18:00 for the 6 days that the PKG was worn.

Active BKS: The Active BKS (aBKS) refers to the median of the 2-min BKS in the period 09:00–18:00 for all days that the PKG was worn excluding BKS > 40 (inactive and sleep) and OFF wrist. Thus, it differs from median BKS in that it also excludes BKS in the inactivity range as well as those in sleep ranges or when the logger was “off-wrist.”

Moving BKS: Over 6 days, there will be 6 BKS recorded at each data point: one from each day. The 25th, 50th and 75th percentile of these 6 BKS is calculated for each 2 min of the day (e.g., 09:00 in Figure 1B). A weighted moving median of 15 of these percentiles (centered on the relevant time of interest) is then calculated. In the case of the 50th percentile, this is known as the moving BKS (or moving BKS_50_). The 25th and 75th percentiles are known as the moving BKS_25_ and moving BKS_75_ respectively. Sleep and “Off wrist” are removed from estimations of the moving BKS.

DKS: The accelerometry data in an epoch is analyzed to produce dyskinesia score (DKS) for that epoch (Griffiths et al., 2012).

Moving DKS: From a 6 day PKG, there will be 6 DKS recorded at each data point: one from each day. The 25th, 50th and 75th percentile of these 6 DKS is calculated for each 2 min of the day. A weighted moving median of 15 of these percentiles (centered on the relevant time of interest) is then calculated. In the case of the 50th percentile, this is known as the moving DKS (also moving DKS_50_). The 25th and 75th percentiles are known as the moving DKS_25_ and moving DKS_75_ respectively. Sleep and “Off wrist” are removed from estimations of the moving DKS.

PTB: The Percent Time in Bradykinesia is an estimate of the proportion of time that the accelerometry data from each epoch is above target (see below). Its derivation is described in detail elsewhere (Khodakarami et al., 2021) but in brief, the PTB is the number of epochs whose BKS were above a target that approximated 35 MDS-UPDRS III points, expressed as a percent of all the available epochs in that period (Khodakarami et al., 2019, 2021).

Dose Interval: The PKG logger vibrates to reminders the wearer when a levodopa doses is due. The Dose interval is calculated from the interval between dose reminders.

### Targets

The therapeutic target for the moving BKS is 26 (Figure 1) and the intention of therapy is to bring the moving BKS below 26 between 09:00 and 18:00. This target for the moving BKS following the advice of senior movement disorder neurologists who were part of the Treat to Target Study Group listed as authors in the original study (Woodrow et al., 2020). The target for PTB is 30%, being the 75th percentile of controls (Khodakarami et al., 2021).

### Subject Inclusion and Case Categorization

#### Study Description

Approval to use and re-analyze data from subjects who participated in a previously published study (Woodrow et al., 2020) was provided by the St Vincent’s Health (Melbourne) Human Research and Ethics Committee. The original study was designed to compare the outcomes of PwP managed by usual care (PKG- arm) with PwP whose treatment was guided by information from the PKG as well as clinical assessment (PKG + arm) (Woodrow et al., 2020). Criteria for inclusion was idiopathic PD in people aged between 59 and 75 years, with a MOCA > 21, with either four or more years of disease or receiving four or more doses of levodopa per day, having no contraindications to increase dopaminergic therapy and being willing to accept changes to their dopaminergic therapy according to the protocol. A previous pilot study (Farzanehfar et al., 2018) showed that contraindications to increasing levodopa were common in people over 75 and they were excluded with the intention of reducing the number of participants who failed to complete the study. Participants referred for device assisted therapy withdrew in the original study because of the practicalities of optimizing the device assisted therapy within the study and as there is a very low threshold in Australia for referring PwP < 59 years for DBS, this age group was excluded because many would not complete the study. End points were improvements in scores from clinical scales (MDS-UPDRS III, MDS-UPDRS-total and PDQ39) administered on entry and exit from the study. The scores from these scales were not available to treating doctors. The 154/200 enrolled participants who completed the original study are the focus of this current study.

As the aim of the study was to test whether outcomes for PwP were improved when doctors used information provided by the PKG, it was important that the experience in PD management in the two arms was similar. Advanced neurology trainees (8 doctors) or recently made Fellows (7 doctors) of the Royal Australasian College of Physicians participated. Experience in PD ranged from 1 to 2 years but slightly more experience in the PKG- arm. All attended 1 day of training in the assessment and management of PD that focused on fluctuations, non-motor features, contraindications to and side effects of anti-Parkinson’s medications, and criteria for device assisted therapies. Doctors in the PKG + arm received a further day of training in interpreting the PKG. All doctors worked at centers specializing in movement disorders, but some also attended regional clinics. Clinics that use the PKG extensively became the PKG + clinics whereas clinics where fellows had infrequent experience with the PKG became PKG- clinics. The study was conducted at 12 clinics (7 in major cities, 3 in regional centers (2 affiliated with a major city service), equally divided between the PKG + and PKG- arms. A median of 12 PwP (IQR = 6.5) attended each site, with each doctor seeing an average of 8 PwP. Few study participants attend participating clinics for their usual care and were reluctant to travel across town or to another town, so they were allocated to the most conveniently located clinic.

**FIGURE 2:**
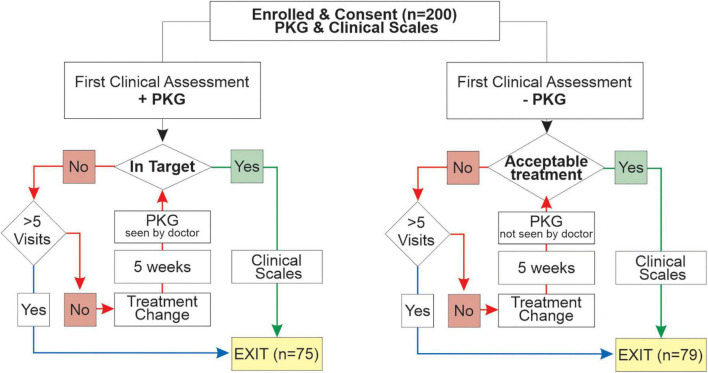
This is a flow chart showing the structure of the original study (Woodrow et al., 2020).

At the first visit, participants in the PKG + arm were assessed using history, examination and the PKG and PKG report to decide whether motor features were in or out of target (Figure 2). PKG + doctors were required under most circumstances to follow the PKG findings in deciding whether to change treatment (see below), noting that always it is the doctor’s clinical judgment as to what treatment decisions were made (including the decision to treat). If the PD was found to be adequality treated and the PKG was “in target,” the PwP exited the study and clinical scales were performed. If a change in treatment was prescribed, a new appointment in no more than 5 weeks’ time was arranged to reassess the PD state, prior to which a second PKG was performed (Figure 2). When control was achieved, this was designated the Final Visit and clinical scales were performed. A very similar process was followed in the PKG- arm except that the although the PKG was performed at every visit, neither it nor the report was available to the doctors (Figure 2). Doctors were allowed a maximum of 5 visits to achieve control. The median number of visits was 3.0 (median 3.2, min 1, max 5) in the PKG + arm and in the PKG- arm the median was 2.0 (median 2.3, min 1, max 4). At the First and Final Visit several clinical scales were performed. Relevant to this study were: Movement Disorder Society UPDRS (MDS-UPDRS) scales; Parkinson’s Disease Questionnaire (PDQ-39), Severity of predominantly Non-dopaminergic Symptoms in PD (SENS-PD) scale (van der Heeden et al., 2016).

The study protocol required doctors in the PKG + arm to follow the PKG findings when deciding whether to change treatment unless:

• In the doctor’s view the PKG was incorrect (e.g., cervical dyskinesia was present, which was not be detected by the PKG). In these circumstances the doctor should manage according to their clinical judgment and either not treat (exit the study) or change therapy (initiating to a follow-up PKG and assessment.
• A contraindication to changing dopaminergic therapy has been identified, including a reasonable concern that it will be induced by a change in therapy. If this happened at the first visit, then the subject left the study having failed eligibility criteria. If a change in therapy had been possible at previous visits, then the subject exited the study with the view that the best possible clinical improvement had been obtained.
• A referral for device assisted therapies was indicated, in which case the participant is unable to complete the study and are referred to the relevant clinical service.
• PwP declines further change. As consent was given to change dosage according to protocol, this was a failed eligibility criteria if it occurred at the first visit. If this occurred at a later visit the doctor was required to attempt reasonable persuasion to agree to changes (noting that an inclusion criterion was an agreement to increase levodopa according to the protocol) and the subject exited the study with the view that the best possible clinical improvement had been obtained.
• Futility: Attempts at earlier visits to improve scores have failed. The subject exited the study with the view that the best possible clinical improvement had been obtained.
• All five visits have been used. The subject exited the study with the view that the best possible clinical improvement had been obtained.

Note that the decision of whether or not to change therapy was always the doctor’s and not the PKG’s. The last five dot points also applied to PKG- doctors.

#### Parkinson’s KinetiGraph Report and Fluctuator Classification

Every PKG was classified by two experienced movement disorder specialists who had no knowledge of the treating clinician’s findings, the participant’s state or the PKG arm. The reporting was qualitative but differed from the technical reports released by the PKG’s manufacturer in being directed at identifying (a) whether or not the moving bradykinesia and dyskinesia scores were above target for any part of the day; (b) whether there was a levodopa response and whether this was supported by reduction of tremor with levodopa and re-emergence with wearing-off; (c) the duration of the levodopa response relative to the dosing intervals; (d) the presence of increase day time sleepiness which may alert to apathy or orthostatic hypotension and: (e) the likely presence of artifact and whether this interferes with the confidence in the PKG interpretation. There was no recommendation regarding choice of medication.

The report also provided an 8-point classification relating to whether the moving bradykinesia and dyskinesia scores were in target range or whether there were fluctuations and/or dyskinesia or non-fluctuating bradykinesia. Cases classified as have peak dose dyskinesia (without or with only minor bradykinesia), with dyskinesia throughout the day (global dyskinesia) or with prominent tremor but controlled bradykinesia were not included in this study because the numbers were too small in any one category to make meaningful analyses. The classification used here closely follows those categories related to bradykinesia, although the terminology has changed (and some have merged) to provide clearer understanding of the relationship between levodopa response and targets. Of the 154 subjects who completed the study, 107 fell into one of the four fluctuator categories described below with the remaining 47 belonged to the other 4 excluded categories (above). Evidence on the PKG of responsiveness to levodopa or wearing-off at least one dose was necessary to identify Fluctuators (F) whereas a Non-fluctuator (NF) classification was where a levodopa response was not apparent on any dose on the moving bradykinesia score (Figure 1). These two categories were further subdivided according to whether their moving bradykinesia score were in target or “Controlled” or were outside target or “Un-controlled” (Figure 1A). The classification in this study is described in detail below and in Figure 1.

##### Non-fluctuator Uncontrolled

These are PwP whose moving bradykinesia score on the PKG was above target throughout the day (Un-controlled) without a levodopa response or wearing-off (Figure 1B). These participants are presumably either undertreated or unresponsive to levodopa. This was classified “Global bradykinesia” in the original study.

##### Fluctuator Uncontrolled

These are PwP whose PKG showed the moving bradykinesia score above target throughout the day (Un-controlled) but do have a levodopa response or wearing-off (Figure 1C). The levodopa response did not lower the moving bradykinesia score sufficiently to enter target. This was classified “Global bradykinesia with wearing-off” in the original study.

##### Fluctuator Controlled (F-C)

These are PwP where the PKG’s moving bradykinesia score was predominantly in target (Controlled) except for periods of wearing-off prior to each dose (Figure 1D). This was classified “Bradykinesia only as wearing-off” or “predominantly bradykinesia with peak dose dyskinesia” in the original study.

##### Non-fluctuator Controlled (NF-C)

These are PwP where the PKG’s moving bradykinesia score is within target throughout the day (Controlled). While usually there is no evidence of fluctuations, there may be periods of wearing-off that do not rise above target (Figure 1E). This was classified “In Target” in the original study. In many circumstances in medicine, bringing scores into a target range is in effect bringing the measured parameter close to some normal or physiological range. The PKG was developed against controls and targets represent an upper limit of scores found in non-PD age matched controls. This category was used to compare the outcomes of subjects whose PKG scores were in target at the final visit with those of participants whose scores were above target at that visit.

The PKG reporter classification of fluctuators was used for the analyses of outcomes to treatment. Using the concordance between whether or not doctors treated PwP whose PKG was report as “in target” as an index, there was high concordance in the PKG + arm (85%) and modest in the PKG- arm (52%) (Woodrow et al., 2020). Of PwP whose PKGs were reported as “out of target,” 12% were not treated in the PKG + arm, whereas 30% were not treated in the PKG- arm. This difference in concordance is to be expected as the PKG + doctors had the PKG report whereas the PKG- doctors did not.

### Statistical Methods

Unpaired *t*-test were used to compare two groups with different subjects, and one-way ANOVA to compare more than two groups with different subjects. Paired *t*-test was used to compare same subjects before and after treatment intervention. Chi-Square test was performed to compare medication choices in PKG + vs. PKG- group. *P*-values of < 0.05 were considered statistically significant. GraphPad Prism software version 8.0 was used.

## Results

### Clinical Scales and Parkinson’s KinetiGraph Measures of Fluctuator Categories at the First Visit

At the first visit in the study (prior to changing therapy), 107/154 participants who completed the original study, met the criteria for one of the four fluctuator categories listed above (Table 1: PKG + and PKG- arms combined). The participants in each category were of similar age and disease duration (*p* = 0.7 and 0.4, ANOVA, respectively), although disease duration of “non-fluctuator uncontrolled” (NF-U) category tended to be shorter.

**TABLE 1:** Comparison of clinical and PKG scores before and after treatment optimization in fluctuator subclasses including cases from both PKG + and PKG- arm.

Clinical Scales	Visit	NF-C (*n* = 33)	F-C (*n* = 28) mean (SD)	*p*§	F-U (*n* = 22) mean (SD)	*p*§	NF-U (*n* = 24) mean (SD)	*p*§
Age		68 (4.4)	68 (4.5)	n/a	67 (4.9)	n/a	68 (5.6)	n/a
Disease duration		5.8 (3.5)	6.3 (4.3)	n/a	5.9 (3.3)	n/a	4.8 (2.7)	n/a
LEDD	First	685 (328)	754 (404)	0.0002	617 (275)	0.0001	612 (280)	0.003
	Last	n/a	897 (478)		893 (315)		914 (445)	
D2	First	75 (100)	79 (116)	0.01	70 (109)	0.06	79 (127)	0.1
	Last	n/a	101 (111)		107 (109)		108 (111)	
MDS-UPDRS I	First	12 (6.2)	11 (5.4)	0.004	13 (5.4)	0.2	10 (5)	0.6
	Last	n/a	8.6 (4.1)		12 (5.7)		11 (5.1)	
MDS-UPDRS II	First	10 (6.5)	10 (6)	0.02	13 (5.2)	0.2	11 (6.3)	0.7
	Last	n/a	8.6 (5.6)		12 (6)		11 (7.1)	
MDS-UPDRS III	First	31 (8.7)	37 (11)	0.0002	41 (8.5)	0.008	39 (9.8)	0.0004
	Last	n/a	32 (9.2)		35 (11)		32 (9.3)	
MDS-UPDRS IV	First	4.6 (3.4)	4.5 (3.7)	0.1	5.6 (3.4)	0.0007	4.8 (3.6)	0.02
	Last	n/a	3.4 (2.4)		3 (2.6)		3.2 (3)	
Total MDS-UPDRS	First	59 (19)	62 (19)	0.0001	73 (13)	0.0009	68 (14)	0.001
	Last	n/a	53 (17)		63 (17)		57 (17)	
PDQ39	First	28 (18)	27 (19)	0.005	32 (16)	0.002	31 (19)	0.3
	Last	n/a	22 (20)		26 (14)		28 (17)	
SENS-PD	First	12 (4.5)	11 (5.1)	0.4	14 (4.8)	0.01	12 (6.9)	0.2
	Last	n/a	11 (5)		12 (4.7)		11 (5.8)	
Active BKS	First	20 (2.8)	24 (2.3)	0.0001	28 (3.8)	0.0001	28 (4.1)	0.007
	Last	n/a	21 (3.4)		25 (4.1)		26 (4.8)	
Percent Time in Bradykinesia	First	25 (12)	51 (18)	0.0001	69 (19)	0.0001	75 (20)	0.0004
	Last	n/a	34 (19)		57 (2.1)		58 (26)	
Number of doses	First	4.5 (1.2)	4.3 (0.7)	0.1	3.8 (0.8)	0.02	3.8 (1.1)	0.0005
	Last	n/a	4.5 (0.8)		4.3 (0.8)		4.5 (0.8)	
Mean dose interval	First	4.2 (1.1)	4.3 (0.8)	0.5	5.2 (2.7)	0.05	5.5 (3.2)	0.008
	Last	n/a	4.1 (2)		4.2 (1)		3.7 (0.7)	

*The “Visit” Column indicates which scores are from the first visit and the last visit for each score. In the original study many NF-C cases are regarded as in target and not requiring treatment: thus, there are no post optimization scores. § p values were obtained using a paired t-test.*

The fluctuators categories stratified participants into increasing severity of disease according to clinical scales and that treating people with fluctuations improved MDS-UPDRS III, Total and PDQ39 to similar levels to subjects who were controlled non-fluctuators at the start of the study. The MDS-UPDRS III and Total scores of controlled non-fluctuators (NF-C) were lower than those of the other 3 categories (*p* = 0.002 and 0.02 respectively, ANOVA).

In terms of PKG parameters, the clearest differences between categories were the percent time above bradykinesia target (PTB), which progressively increased across categories from subjects in target (NF-C) to those who were out of target and non-fluctuators (NF-U) (*P*-value 0.0001, ANOVA). The PKG’s active bradykinesia score (aBKS) followed a similar pattern to the MDS-UPDRS III. Note that subjects requiring advanced therapy were referred out of the original study.

### Changes in Clinical and Parkinson’s KinetiGraph Measures Following Attempts to Optimize Therapy

At the final visit, treating clinicians considered therapy to have been optimized, using clinical judgment in the case of the PKG- arm or as close to target as clinically possible using the PKG + arm. Scores at final visit were compared with first scores (Table 1). As the PKG scores of controlled non-fluctuators (NF-C) were already in target at first visit these cases were not included in this analysis. In the other 3 categories, all scores from clinical scales improved by the last visit, and many were statistically significant. The MDS-UPDRS III and Total scores improved with changes that are in the moderate to large clinically meaningful range (Shulman et al., 2010). There were also significant changes in the PDQ 39 scores and more modest improvement in SENS-PD scores. Changes in MDS-UPDRS I, II, IV were modest and probably not clinically meaningful. The PKG’s active bradykinesia score (aBKS) and percent time above target (PTB) also changed significantly. The PTB scores remained above 30% (upper limit of normal), suggesting that fluctuations were not completely resolved, especially in uncontrolled fluctuator and non-fluctuator categories (F-U and NF-U).

### The Impact of Objective Measurement on Clinical Outcome

In the 3 treatable categories [i.e., excluding those in target at first visit and without fluctuations: NF-C category (*n* = 33)], there were 37 in each arm (Figure 1A and Table 2). The MDS-UPDRS III, Total and PDQ39 scores were not significantly different in the two arms (*p* = 0.35, 0.23 and 0.40 respectively: *t*-test). The difference in MDS-UPDRS III and Total scores between first and last visit in the PKG + arm, were 6.7 and 10.3 (respectively), which lie between moderate and large clinically important differences (Shulman et al., 2010). These differences were significantly larger than the corresponding changes in the PKG- arm (3.7 and 6.7 respectively), which are minimal to moderate clinically important differences. These differences were significantly greater in the PKG + arm (Table 2). The PDQ39 scores were also significantly larger in the PKG + arm (6.8) than in the PKG- arm (2.1 PDQ39 points, Table 2). There were similar differences in the PKG’s bradykinesia score (aBKS) and in the SENS-PD scores.

**TABLE 2:** Change in scores in PKG + arm compared to change in scores in PKG- arm.

Scale/Score	PKG + Mean (± SD)		PKG- Mean (± SD)		Mean Δ (± SD)	95% CI	Effect size	*p* val.
	1st visit	1st -final visit	1st visit	1st -final visit				
MDS-UPDRS III	37.8 (9.2)	8.5 (8.4)	40.0 (10.4)	3.7 (6.7)	4.8 (10.3)	8.3 to −1.3	0.46	0.007
MDS-UPDRS Total	64.6 (14.6)	13 (12)	69.3 (18)	6.8 (10)	6.1 (15.8)	11.4 to −0.76	0.39	0.02
PDQ39	27.8 (14.9)	6.8 (12)	31.4 (20.8)	2.1 (7.2)	4.7 (13.4)	9.2 to −0.22	0.35	0.04
SENS PD	11.8 (5.8)	2.4 (4.7)	12.4 (5.7)	0.11 (3.9)	2.3 (6.1)	4.3 to −0.27	0.38	0.02
Active BKS	25.8 (3.5)	3.2 (2.5)	27.3 (4.1)	1.7 (3)	1.5 (3.8)	2.8 to −0.28	0.39	0.01
PTB*	61.4 (20.2)	19 (14)	66.5 (22.7)	12 (17)	6.9 (21.9)	14–0.28	0.32	0.06

Mean Δ refers to difference in the mean scores of the PKG + scores compared to the mean PKG- scores (first visit minus last visit). 95% CI refers to the 95th percentile confidence limits. Effect size was calculated as the difference in the means, divided by the standard deviation. * PTB, Percent Time in Bradykinesia.

#### Changes in Fluctuation Classification Following Treatment

**FIGURE 3:**
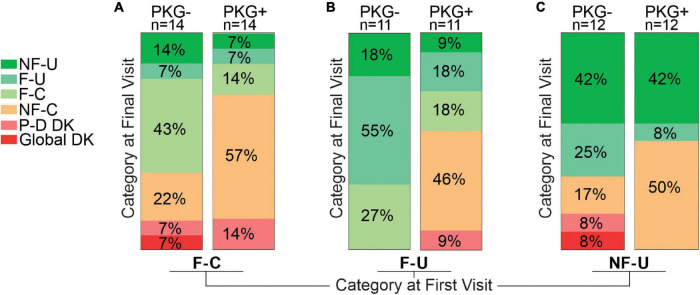
This figure is based on the assumption that the aim of therapy is to convert fluctuators into Non-fluctuators Controlled category or a category as near as possible to controlled. This figure aims to provide a pictographic representation of the relative success in achieving this for subjects who, at the start of the study, were in one of the three “uncontrolled” categories, and subgrouped according to whether they were in the PKG- or PKG + arm. Panel **(A)** shows the 28 participants who were in the Fluctuator–Controlled (F-C) category at the start of the study with those in the PKG- arm depicted by the right Column and those in the PKG + arm by the left column. Each column is divided into segments whose color represents a fluctuator state, with the legend on the left of the figure, where P-D DK represents “Peak dose dyskinesia” and Global DK represents “global dyskinesia” or dyskinesia throughout the whole day. The size of each colored segment and the percentage within each segment of the column indicates the proportion of participants in that color coded category at study end. Panel **(B)** is a similar plot for participants who were in Fluctuator –Uncontrolled (F-U) at the start of the study. Panel **(C)** is a plot of the outcome of participants who were in Non-fluctuator –Uncontrolled (NF-U) at the start of the study.

The 74 cases that were not in target (i.e., excluding NF-C) at the outset of the study were sorted into the fluctuator state that they attained by the end of the study and compared with the fluctuator category at first visit (Figure 3 and Table 3).

**TABLE 3:** Changes in Clinical according to the fluctuator category reached at end of study.

Score	NF-C *N* = 24	F-C *N* = 13	F-U *N* = 14	NF-U *N* = 16	DK *N* = 7
ΔMDS-UPDRS III	8.5 (7.4)****	6 (9.6)*	1.5 (5) n.s.	6.6 (7.5)**	4.7 (9.5)n.s.
ΔMDS-UPDRS IV	2.2 (3.1)**	1.3 (3) n.s.	2.8 (4.5)*	1.5 (4) n.s.	−0.4 (4.3) n.s.
ΔMDS-UPDRS Total	13.5 (11.3)****	9.6 (13.5)*	4 (7) n.s.	9.5 (11.5)**	8.8 (15.6) n.s.
ΔPDQ39	6.6 (13.8)*	7.3 (6.2)**	1.2 (7.2) n.s.	2.7 (5.7) n.s.	1.1 (9.6) n.s.
ΔSENS	2.5 (4.5)*	0.07 (3) n.s	1.5 (2.5)*	0.9 (5) n.s.	−0.8 (7) n.s.
Δ Active BKS	3 (2.4)****	2 (1.5)***	1.9 (2.6)**	1.1 (3.1) n.s.	−5.5 (3.3)**
ΔPTB	18.7 (17.6)****	17.3 (13)***	10.4 (12.4)**	6.1 (11.2)*	30.4 (15.6)**
ΔLEDD	−200 (278)***	−267 (144)****	−188 (180)**	−340 (453)**	−148 (185) n.s.
ΔD2	−64.5 (82.4)***	−26 (56) n.s.	−16 (31) n.s.	15 (94) n.s.	−21.4 (36.6) n.s.

Δ = value of score at first visit—score at last visit, n.s. = p > 0.05, *0.05 > p > 0.01, **0.01 > p > 0.001, ***0.001 > p > 0.0001, ****0.0001 > p > 0.00001. P-values were obtained using the Paired t-test. Values are mean and SD.

##### Fluctuator Controlled Category at First Visit

Totally 11 (8 in PKG + arm) of 28 entered the target range (NF-C) a small proportion deteriorated (F-U or NF-U) or developed dyskinesia. Despite the increased use of dopaminergic agents (Table 3) there was a significant 2.2 points fall in MDS-UPDRS IV.

##### Fluctuator Uncontrolled Category at First Visit

Totally 46% in PKG + arm became controlled compared to no cases in the PKG- arm.

##### Non-fluctuator Uncontrolled Category at First Visit

Totally 50% became controlled in the PKG + arm compared to 17% in the PKG- arm. The UPDRS III and Total scores of these subjects improved significantly (Table 3) in association with the greatest increase in LEDD. As these cases were “non-fluctuators” without short duration levodopa responses, it is possible these cases were undertreated and had not yet having developed wearing-off. However, disease duration was 3 years or more in 75% (Table 1). In both arms, 42% remained “non-fluctuators” (NF-U, Figure 3C) at the final visit and treating doctors noted that contraindications prevented adequate increase in therapy in some cases.

Acquiring the PKG target range (33%, 19/24 coming from the PKG + arm) provided the largest changes in MDS-UPDRS III and Total scores (Table 3) and a satisfactory MDS-UPDRS III (27, SD 8.8). Changes in PDQ39 and SENS-PD were also largest (and significant) in participants who were in NF-C or F-C categories at the final visit. Those whose PKG scores improved but failed to enter target (F-U or NF-U) at study end generally had smaller and/or non-significant changes. Dyskinesia, mainly peak-dose dyskinesia, emerged in 6/74 participants but were too few to provide statistical power for analysis.

### Changes in LEDD Required to Produce Improvement in Scores

At the first visit, the LEDD in the PKG + arm was significantly less than in the PKG- arm. There was a larger change in the LEDD from first to final visit in the PKG + arm than in the PKG- arm and the difference was in part due to a substantial contribution for an increase in D2 agonists (Table 4). This is difficult to interpret because the increased LEDD in the PKG + arm had the result of producing similar LEDD in both arms at the final visit. The most common practice used to treat fluctuators was to shorten the levodopa dose interval. In the PKG- arm this was accompanied by increasing the levodopa dose, whereas clinicians in PKG + group were more likely to add and/or increase the dose of D2 agonists (Chi-square test, *p*-value = 0.005). This is apparent in Table 3, which shows that largest change in D2 agonists were observed in participants who achieved target at the end of the study, representing more than 25% of the total increase in D2 agonists. These were mainly subjects in the PKG + arm. On the other hand, 69% of subjects who finished the study in the F-U category were from the PKG- arm, and D2 agonists constituted 9% of the average LEDD used. It is also noteworthy that the largest increase in LEDD were in those who remained in the NF-U category, whereas those who developed dyskinesia had the smallest increase in LEDD, possibly because their first visit scores were already close to optimal.

**TABLE 4:** Changes in LEDD in PKG + and PKG- arms.

	PKG +		PKG-	
	
	Median (IQR)	Δ median	Median (IQR)	Δ median
LEDD 1st Visit	500 (320)	300	638 (416)	201
LEDD Final Visit	800 (469)		839 (425)	
D2 1st Visit	0 (75)	113	0 (165)	44
D2 Final Visit	113 (125)		44 (150)	

*LEDD, Levodopa Equivalent Daily Dose; IQR, Interquartile range; Δ median, change in median value from first visit to final visit.*

Despite the increased use of dopaminergic agents in those subjects who reached target, there was 2.2 points fall in the MDS-UPDRS IV, which was significant (Table 3). Overall, the reduction in MDS-UPDRS IV points in the PKG + arm was greater than in the PKG- arm.

## Discussion

The aim of this study was to assess whether the use of sensor data to identify fluctuations and to guide adequacy of treatment relative to targets provided better outcomes in terms of clinical scales than conventional management of fluctuations. These aims were addressed by showing the following:

• Fluctuators categories stratified participants into increasing severity of disease as measured by clinical scales (Table 1). Treating subjects so that their fluctuator category was less severe improved scores from clinical scales (Figure 3). Together this provides face validity of the classification. The relationship to the clinically recognized phenomena is discussed further below.
• Scores from clinical scales, including quality of life scores, were significantly better when identification and treatment of fluctuations was aided by objective measurement.
• Outcomes, including non-motor scores and quality of life scores, were greatest in those subjects whose PKG scores could be brought into the target range. When the PKG was used, the severity of fluctuator category at the onset of the study did not influence the likelihood of achieving target.
• The use of objective monitoring led to greater use of D2 agonists and more frequent dose of levodopa with a non-significantly trend to use a larger LEDD. This did not increase UPDRS IV scores.

### Relationship of Fluctuator Categories to Clinically Recognized Wearing-Off

The return of symptoms prior to the next levodopa dose (Stacy, 2010) and an implied levodopa response (Chou et al., 2018) are central to the clinical description of “wearing-off.” In the training for the original study (Woodrow et al., 2020), clinicians in both arms were encouraged to use questioning described in wearing-off questionnaires (Stacy et al., 2008; Stacy, 2010) to establish the presence of wearing -off and whether there was a levodopa response. As shown in Figure 1, the fluctuator category also required a levodopa response and/or wearing off detected through the PKG measurement, that lead to some, or all of the day having unsatisfactory bradykinesia (out of target) and is thus based on the type of information that a practicing clinician would seek when making managing fluctuations. The validity of the PKG criteria was supported by the MDS UPDRS III, MDS-UPDRS Total and PDQ39 scores which increased as the fluctuator category moved increasingly out of target. Furthermore, clinical scores improved the most in the study arm with the greatest number of cases in target (PKG + arm) and those cases that moved into target acquired better clinical scores than those whose best outcome was furthest from target (Figure 3 and Table 3). These changes in clinical score provide some validity to the fluctuator categories.

The original study did not use either wearing-off scales or diaries, so it is not possible to compare these measures with the PKG categories. However, both measures report symptomatic experience, which while relevant and important, is not the same as an objective measurement. Thus, the question here is not how much the PKG scores and wearing-off scales or diaries might have differed, but that supporting therapy decisions with objective measurements (PKG + arm) led to better outcomes than using the same symptomatic reporting embedded in wearing off scales and the diaries to guide clinicians (PKG- arm).

### The Use of a Target Range

Treatment of fluctuations requires two important pieces of information. The first is knowledge about the duration of benefit of levodopa, which is relevant to the optimal dosing interval of levodopa. The second important piece of information is whether the response has reduced bradykinesia to an acceptable level without excess dyskinesia (i.e., in target). This is crucial for arbitrating between decisions to increase the dose of levodopa (separate to shortening the dose interval): if the best response is in target but dose interval is too long, then avoiding an increase in dose will also avoid peak dose dyskinesia. It may point to the use of D2 agonists over levodopa. The relative accuracy of this information obtained by history compared to PKG may be a reason for the difference in results in the two arms.

The concept of a target or therapeutic range is widely used in medicine and similarly, a target range has been proposed for the PKG (Odin et al., 2018; Pahwa et al., 2018). Managing PD to this target resulted in improved outcomes (Woodrow et al., 2020) with an the average MDS-UPDSR III score of 28.6 in the PKG + arm at the end of that trial (c.f 27 (SD 8.6) in fluctuators converted into target (NF C) in this study). The results suggest that clinicians in the PKG- arm, lacking objective data, made smaller therapeutic interventions and so were less likely to bring scores into target (and more likely to produce dyskinesia). The validity of the PKG criteria was supported by increasing MDS UPDRS III, MDS-UPDRS Total and PDQ39 scores as fluctuator category moved increasingly out of target. Furthermore, cases in target at study end had the most improvement in clinical scores (Table 3). These changes in clinical score provide some validity to the fluctuator categories.

### The Implications of Increasing LEDD

It is unlikely that the difference in use of D2 agonists and the change in LEDD in the 2 arms was due to a different knowledge base or experience of clinicians in the two arms as the same training program was given to both arms prior to commencing the study and clinicians in both arms were at a similar stage in their training as fellows (Woodrow et al., 2020). It is possible that the explanation lies in the training program, which advised clinicians in both arms to avoid further increase in the size of each dose but instead shorten levodopa dose interval or add a long acting D2 agonists when the best response was in or near target. We suspect that this calculation was easier to make when PKG graph was available for inspection. This explanation would explain both the difference in deployment of D2 agonists and levodopa as well as the avoidance of significant dyskinesia while increasing the LEDD. Doctors in the PKG + arm required more visits to achieve the optimum LEDD (median 3.0 v 2.0 in PKG-arm *p* < 0.0001, *t*-test). This is most likely due to doctors making similar increments in dopaminergic agents at each visit and so more visits are required if a larger change in LEDD were to occur. The UPDRS IV reduced in both arms despite increases in the LEDD.

### The Validity of Non-fluctuator Uncontrolled as a Fluctuator Classification

While the non-fluctuator Uncontrolled (NF-U) classification is not a fluctuator, it was included because these cases may have been sufficiently undertreated that a levodopa response was not apparent. Figure 3 and Table 3 suggest that most (58%) did change fluctuator category after increasing dopaminergic stimulation (i.e., they became fluctuators). However, the remaining 42% remained in the NF-U category suggesting that they were indeed true non-fluctuators. It is unlikely that they were cases of early PD who had not yet developed shortening of the levodopa response, because they should still have been able improve their bradykinesia scores and become Controlled Non-fluctuators. The proportion was similar in both arms suggesting there were common factors beyond the recognition of fluctuations. The inclusion and exclusion criteria for the original study (Woodrow et al., 2020) were designed to increase the proportion of participants who were both responsive to levodopa and without contraindication to increase in dopaminergic agents. Nevertheless, contraindications to increase in dopaminergic agents was a factor in a proportion and it is possible that some cases may not have been idiopathic PD. It is also possible that varying response to levodopa (e.g., caused by impaired gastric emptying) may have contributed in some of these cases. PwP find response and duration of response to levodopa difficult to assess and provide an accurate history when delayed gastric emptying is present. As well the summary plot of the PKG is derived from averaging of 6 days and is thus most effective in showing levodopa responses that are consistent and reliable. While it is possible to detect clues to delayed gastric emptying from the PKG it does take more experience than was the case with participating clinicians. This highlights the need for better ways of extracting information about the presence of delayed gastric emptying in individual subjects. The inclusion criteria for this study were designed to recruit predominantly subjects in whom fluctuations had developed and to exclude subjects with orthostatic hypotension. Thus, subjects with significant autonomic dysfunction, including gastroparesis may be underrepresented compared to the whole range of the PD populations. Subjects who met the criteria for advanced therapies were referred out of the study and will also be underrepresented. However, this study was not intended to measure the incidence of fluctuations: rather it was intended to assess the utility and benefit of using an objective measure to identify and treat fluctuators.

### Quality of Life in Fluctuators

The severity of fluctuations affects quality of life (as measured by PDQ39). This conclusion is drawn from noting that PDQ39 increases progressively (although not significant statistically) as the category of fluctuation moves further from being in target, that treating fluctuators leads to improved PDQ39 scores and that subjects who were in target (NF-C) or near target (F-C) at study end (Table 3) had the largest change in PDQ39. Changes in bradykinesia are a marker of changing dopaminergic transmission, regardless of whether the PwP experiences it as motor or non-motor. It is thus likely that those non-motor symptoms responsive to dopaminergic transmission may also improve using objectively measured bradykinesia as a marker of the state of dopaminergic transmission. While SENS-PD scores were similar in each fluctuator category at first visit (Table 1), the SENS = PD scores improved significantly in the PKG + arm, especially in those that reached target (Tables 2, 3).

This cohort had relatively low PDQ39 scores, possibly because subjects with contraindications to increasing levodopa were excluded both directly as well as indirectly, by limits on upper age (75 years) and MoCA (< 21). As people aged over 75 and with low MoCA scores had improved PDQ39 scores when management was assisted with objective measurement and a target range (Farzanehfar et al., 2018), it is likely that objective measurement would be helpful in those cases who were excluded from this study. The exclusion of subjects who were eligible for device assisted therapies may also be relevant. It is very likely that objective measurement in conjunction with the use of a target range would be helpful in identify people who would benefit from device assisted therapy and in optimizing the therapy once implemented: the authors’ experience supports this. Nevertheless, future studies are required to confirm whether this is the case.

The PKG system only records upper limb motion and does not provide indication of cranio-cervical or axial dyskinesia. Although this was not a major factor in this study because predominantly dyskinetic subjects were not included in the fluctuator classification, this is a limitation when used to assess fluctuations when dyskinesia is also present. The PKG does not report axial rigidity, axial bradykinesia or Freezing of Gait. These are important limitations but may not have greatly influenced this study because the exclusion criteria of a MoCA < 21 would exclude many PwP with Freezing of Gait.

## Conclusion

This study demonstrates that objective measurement may help clinicians to identify and classify the severity of fluctuations in PwP. Treating these subjects to target range results in more subjects having fully treated fluctuations and improve clinical scales including PDQ39 scores. The use of sensor measurement in routine clinical practice will require the capacity to extract the information provided by the sensor, either by the clinician themselves or by an expert reporter (as occurs with an ECG). This skill is relatively easy to learn and was delivered to the PKG + doctors in a day’s training. However, more accurate information about the nature of fluctuations is only one side of the therapeutic response: training in the appropriate use of therapeutic agents may also be required in routine clinical practice. It is possible that the improvement in the PKG- arm was due to the 1-day training in the treatment of fluctuations. If these issues are addressed then objective measurement of bradykinesia scores to treat to a target range is likely to particularly assist non-specialist clinicians, such as general neurologists and geriatricians, to more effectively manage PwP experiencing fluctuations.

## Data Availability Statement

The data analyzed in this study is subject to the following licenses/restrictions: The data from the original study is available on reasonable request to the corresponding author. Requests to access these datasets should be directed to MH, malcolm.horne@florey.edu.au.

## Ethics Statement

The studies involving human participants were reviewed and approved by St Vincent’s Health (Melbourne) Human Research and Ethics Committee. The patients/participants provided their written informed consent to participate in this study.

## Author Contributions

HW contributed in coordinating subjects and collecting data related to clinical scales. PF did the data analyses. PF and MH wrote the manuscript. All authors reviewed the manuscript.

## Conflict of Interest

Global Kinetics Corporation (GKC) is the manufacturer and distributor of the Parkinson’s KinetiGraph (PKG). MH is an inventor of the PKG system and was Chief Scientific Officer in GKC and also has equity in GKC. HW is supported by a grant provided by GKC to the Florey Institute. The remaining author declares that the research was conducted in the absence of any commercial or financial relationships that could be construed as a potential conflict of interest.

## Publisher’s Note

All claims expressed in this article are solely those of the authors and do not necessarily represent those of their affiliated organizations, or those of the publisher, the editors and the reviewers. Any product that may be evaluated in this article, or claim that may be made by its manufacturer, is not guaranteed or endorsed by the publisher.
